# Supplementary material for: Evaluation in mice of cell-free produced CT584 as a Chlamydia vaccine antigen
Source: bioRxiv. 2024 Jun 6:2024.06.04.597210. Preprint. [Version 1] doi: 10.1101/2024.06.04.597210 (PMC11185655; doi:10.1101/2024.06.04.597210)
Supplement: Supplement 1 [file NIHPP2024.06.04.597210v1-supplement-1.pdf]

bioRxiv preprint doi: https://doi.org/10.1101/2024.06.04.597210; this version posted June 6, 2024. The copyright holder for this preprint (which was not certified by peer review) is the author/funder, who has granted bioRxiv a license to display the preprint in perpetuity. It is made available under aCC-BY 4.0 International license.

Figure S1. *Cm* TC0873 and *Ct* CT584 are nearly identical

A) Smith-Waterman local sequence alignment for *Cm* TC0873 and *Ct* CT584

Figure S2. *E. coli* DNA codon optimization for *Ct* CT584

A) Smith-Waterman local sequence alignment for the native *Ct* CT584 and *E. coli* optimized DNA sequence

bioRxiv preprint doi: https://doi.org/10.1101/2024.06.04.597210; this version posted June 6, 2024. The copyright holder for this preprint (which was not certified by peer review) is the author/funder, who has granted bioRxiv a license to display the preprint in perpetuity. It is made available under aCC-BY 4.0 International license.

Figure S1. Cm TC0873 and Ct CT584 are nearly identical

Smith-Waterman Local Alignment

```
Cm TC0873   1  MTTKSKTLEIDNNTFLLLEGNLKRIFATPIGYTTFREFQNVIFNCAQGQQ   50
Ct CT584    1  MTTKPKTLEIDNNTFLLLEGNLKRIFATPIGYTTFREFQNVVFNCAQGQQ   50

Cm TC0873  51  ELANFLFEMLINGKLLQELPAGQKQSAQSLIVQFMMLIRVAKDIHERGEF  100
Ct CT584   51  ELANFLFEMLINGKLLQELPAGQKQSAQSLIVQFMMPIRVAKDIHERGEF  100

Cm TC0873 101  INFITSDMLAQQERCVFLNRLSRVDGQEFLLMTDVQNTCHLIRHLLSRLL  150
Ct CT584  101  INFITSDMLAQQERCVFLNRLSRVDGQEFLLMTDVQNTCHLIRHLLSRLL  150

Cm TC0873 151  EAQKNPIGEKNLQEVQEDLDSLRAHFEELTKSM  183
Ct CT584  151  EAQKNPIGEKNLQEIQEDLDSLRAHFEELTKSV  183
```

Similar
Not Similar
Identical

bioRxiv preprint doi: https://doi.org/10.1101/2024.06.04.597210; this version posted June 6, 2024. The copyright holder for this preprint (which was not certified by peer review) is the author/funder, who has granted bioRxiv a license to display the preprint in perpetuity. It is made available under aCC-BY 4.0 International license.

Figure S2. E. coli DNA codon optimization for Ct CT584

Smith-Waterman Local Alignment

```
Native CT584       1  ATGACGACGAAACCCAAAACTCTAGAAATCGATAACAACACGTTCCTGCTTTTGGAAGGC  60
Codon Optimized    1  ATGACTACAAAACCCAAGACGCTAGAAATAGACAACAACACCTTCCTGTTGTTAGAAGGT  60

Native CT584      61  AATTTAAAAAGAATTTTTGCCACGCCTATTGGGTATACAACGTTTAGAGAATTCCAAAAT  120
Codon Optimized   61  AATCTGAAACGTATTTTTGCGACTCCAATCGGCTACACGACCTTTCGTGAATTCCAAAAC  120

Native CT584     121  GTAGTGTTTAACTGCGCACAAGGACAACAGGAGCTTGCAAATTTTTTATTTGAGATGTTG  180
Codon Optimized  121  GTGGTGTTCAACTGCGCCCAAGGTCAACAAGAGTTGGCTAATTTCTTGTTTGAAATGCTG  180

Native CT584     181  ATCAATGGGAAGTTGTTGCAAGAGCTACCTGCAGGACAGAAACA-ATCTGCACAAAGTCT  239
Codon Optimized  181  ATTAACGGCAAACTGCTGCAAGAGCTGCCGGCGGGCCAGAAGCAGAGC-GCACAATCCCT  239

Native CT584     240  AATTGTACAATTTATGATGCCGATTCGTGTTGCGAAGGATATCCATGAGCGCGGTGAATT  299
Codon Optimized  240  GATTGTACAGTTTATGATGCCGATCCGCGTGGCCAAAGATATTCATGAACGCGGTGAATT  299

Native CT584     300  CATCAATTTCATTACTTCCGATATGCTAGCTCAACAGGAACGCTGCGTATTTTTAAACCG  359
Codon Optimized  300  TATCAACTTCATCACCAGCGACATGCTGGCGCAACAGGAGCGCTGTGTTTTCTTGAATCG  359

Native CT584     360  ACTCTCTCGAGTTGATGGGCAGGAATTTCTACTCATGACGGATGTACAAAATACCTGCCA  419
Codon Optimized  360  TCTGTCTCGTGTCGACGGCCAGGAGTTCTTGCTGATGACCGATGTTCAGAATACGTGCCA  419

Native CT584     420  TCTCATCCGCCA----CTTACT-GTCTCGTCTGCTTGAAGCGCAAAAAAATCCTATCGGA  474
Codon Optimized  420  TCTAATCCGTCATCTGCTTAGTAGACTC-----CTGGAGGCTCAGAAGAACCCGATTGGT  474

Native CT584     475  GAAAAAAATCTCCAAGAAATCCAAGAAGATCTCGATTCTTTACGCGCTCACTTTGAAGAA  534
Codon Optimized  475  GAAAAGAACCTGCAGGAGATCCAGGAAGATCTGGACAGCCTGCGTGCACATTTTGAGGAA  534

Native CT584     535  TTAACGAAA  543
Codon Optimized  535  TTGACCAAA  543
```

Identical

Different
